# Heel raises versus prefabricated orthoses in the treatment of posterior heel pain associated with calcaneal apophysitis (Sever's Disease): study protocol for a randomised controlled trial

**DOI:** 10.1186/1757-1146-3-3

**Published:** 2010-03-02

**Authors:** Alicia M James, Cylie M Williams, Terry P Haines

**Affiliations:** 1 Cardinia Casey Community Health Service, Southern Health, Cranbourne, Australia; 2 Peninsula Community Health Service - Frankston, Peninsula Health, Frankston, Australia; 3 Allied Health Clinical Research Unit, Southern Health, Cheltenham, Australia; 4 Physiotherapy Department, Monash University, Frankston, Australia

## Abstract

**Background:**

Posterior Heel pain can present in children of 8 to 14 years, associated with or clinically diagnosed as Sever's disease, or calcaneal apophysitis. Presently, there are no comparative randomised studies evaluating treatment options for posterior heel pain in children with the clinical diagnosis of calcaneal apophysitis or Sever's disease. This study seeks to compare the clinical efficacy of some currently employed treatment options for the relief of disability and pain associated with posterior heel pain in children.

**Method:**

Design: Factorial 2 × 2 randomised controlled trial with monthly follow-up for 3 months.

Participants: Children with clinically diagnosed posterior heel pain possibly associated with calcaneal apophysitis/Sever's disease (n = 124).

Interventions: Treatment factor 1 will be two types of shoe orthoses: a heel raise or prefabricated orthoses. Both of these interventions are widely available, mutually exclusive treatment approaches that are relatively low in cost. Treatment factor 2 will be a footwear prescription/replacement intervention involving a shoe with a firm heel counter, dual density EVA midsole and rear foot control. The alternate condition in this factor is no footwear prescription/replacement, with the participant wearing their current footwear.

Outcomes: Oxford Foot and Ankle Questionnaire and the Faces pain scale.

**Discussion:**

This will be a randomised trial to compare the efficacy of various treatment options for posterior heel pain in children that may be associated with calcaneal apophysitis also known as Sever's disease.

**Trial Registration:**

Trial Number: ACTRN12609000696291

Ethics Approval Southern Health: HREC Ref: 09271B

## Introduction

Calcaneal apophysitis (also known as Sever's disease [1]) is an overuse syndrome thought to be caused by repetitive micro trauma due to increased traction of the calcaneo-achilles apophysis [1-3]. This condition is characterised by pain experienced near the lower posterior aspect of the calcaneus in close proximity to the attachment of the Achilles tendon into the secondary growth plate of the calcaneus. The calcaneal growth centre or apophysis appears at approximately seven years of age [4] and fuses in girls of age approximately thirteen years and boys of fifteen years [2,5,6], hence this condition is typically seen in pre-adolescent and adolescent children. Posterior heel pain reportedly associated with Calcaneal apophysitis has been reported to comprise 2%-16% of musculoskeletal injuries in children [2,7,8].

Several theories regarding the pathomechanics of posterior heel pain associated with calcaneal apophysitis in children have been proposed and can be categorised into the following:

1. Growth and gastrocnemius/soleus tightness: The presentation of calcaneal apophysitis is thought to be due to a period of rapid growth. The rapid period of growth caused increased relative tension in the Achilles tendon/triceps surae complex which amplifies traction on the apophysis [2,3,9].

2. Biomechanics: It has previously been suggested that children with cavus or planus foot types are more susceptible to calcaneal apophysitis possibly due to a harder heel strike placing increase strain on the affected area [10-12].

3. Infection: Previous authors have reported infection to have directly caused calcaneal apophysitis [10,13], though other authors have listed infection as a differential diagnosis [3,10,11,14].

4. Trauma: Repetitive or single traumatic incidents have been anecdotally reported to be the causes of posterior heel pain in calcaneal apophysitsis [15-17]. There is limited evidence to support this hypothesis.

5. Obesity: In children obesity has been observed as an influential factor in calcaneal apophysitis [1,6,18].

Despite the presence of these theories, there has been limited clinical data presented to support them to date.

Recommend treatment paths for posterior heel pain clinically diagnosed to be associated with calcaneal apophysitis are varied with most publications relying upon earlier study recommendations [19]. Treatment recommendations have included: rest or cessation of sport [3,20,21], use of heel lifts [2,22,23], use of mobilisation [1,2,22], orthoses [21,22,24], stretching or strengthening [20,21,24], padding for shock absorption/strapping of heel [24-26], ultrasound/pharmaceutical prescriptions/ice [20,21,27], immobilisation casting or crutches [23,26,28] or removal of apophysis [29]. A recent literature review concluded that due to no valid or reliable data being available regarding calcaneal apophysitis causation and no clinical trial comparing treatment approaches, no clinical treatment path can be determined as "best practice" [19], therefore further research into treatment options is required.

This study aims to compare two clinically applied treatment options for the management of posterior heel pain associated with the clinical diagnosis of calcaneal apophysitis.

## Method

### Study Design

**Figure 1:**
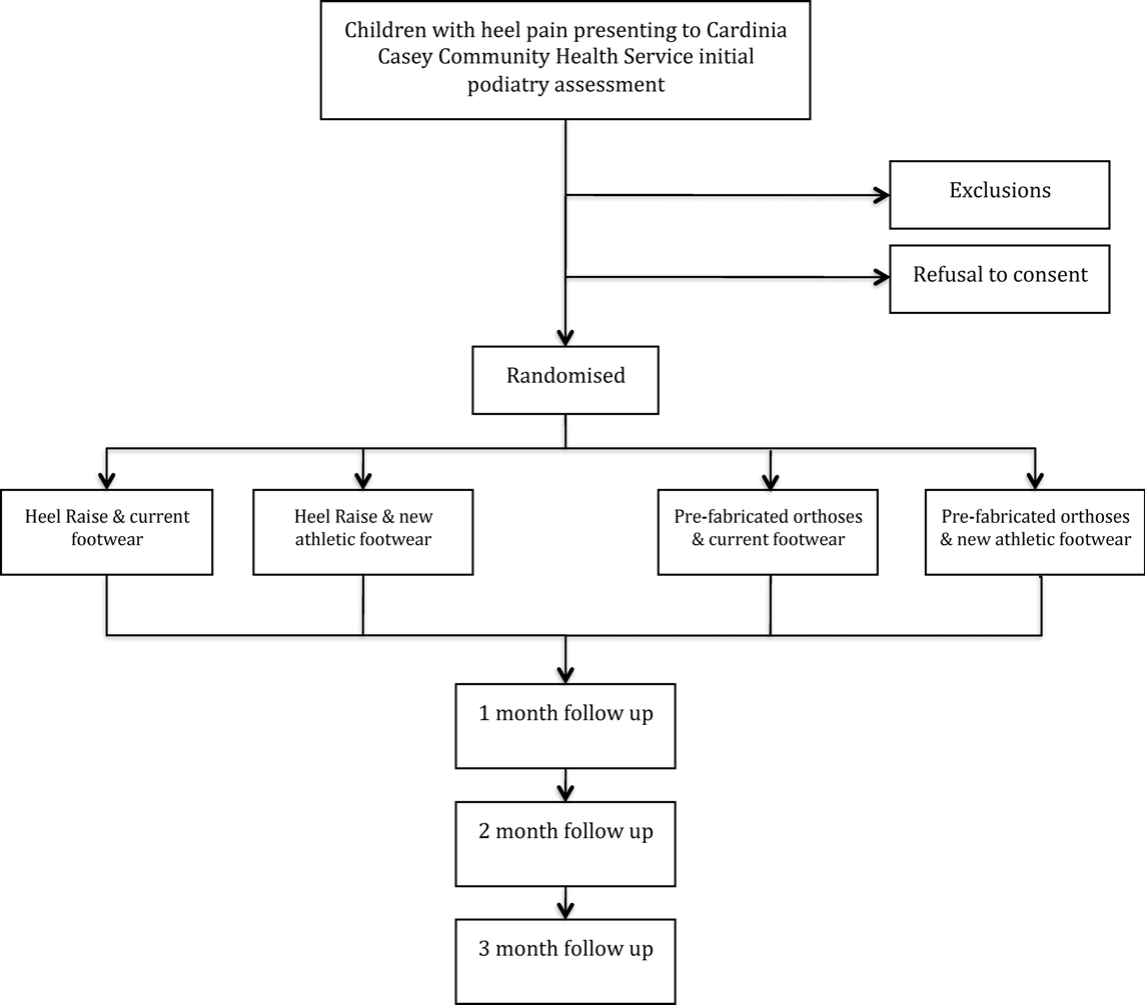
**Consort flow chart for the study**.

This is a factorial randomised controlled trial; two factors (shoe orthosis and footwear) each with two levels (heel raise/pre-fabricated orthoses and current footwear/new athletic footwear respectively), with a three month follow-up period. A consort flow chart for the design of this study is presented (Figure 1). There is no control group due to this clinical trial being conducted within a health setting. The trial is also being conducted within a lower socioeconomic catchment and it is well documented that there is lower participation in sporting activities [30] and higher rates of obesity [31,32] in these areas. It was decided that there was a risk of participant's not re-starting physical activity should there be a cessation of sport group.

### Participants and Setting

Children aged between eight and fourteen years will be recruited from the case load of podiatrists at Cardinia Casey Community Health Service and Peninsula Health Service. Patients will be eligible to participate if they provide a subjective report of pain located at the calcaneal apophysis (i.e., posterior aspect of heel) with pain on palpation (positive calcaneal squeeze medial and lateral borders), have not in the last 12 months been diagnosed fracture or tumour of the foot or leg and have not been diagnosed with infective, reactive or rheumatoid arthritis.

### Interventions

#### Minimum care for all participants

All participants will receive a standardised icing and stretching program. The participants will be asked to ice for 10 minutes a day, during the initial stage of treatment (one month). The icing treatment will continue only after sporting activities until the participant is pain free. The stretching program will be initiated after the acute phase of calcaneal apophysitis. The stretch will be an isometric weight-bearing gastrocnemius stretch.

#### Factor 1: Shoe orthoses

**Figure 2:**
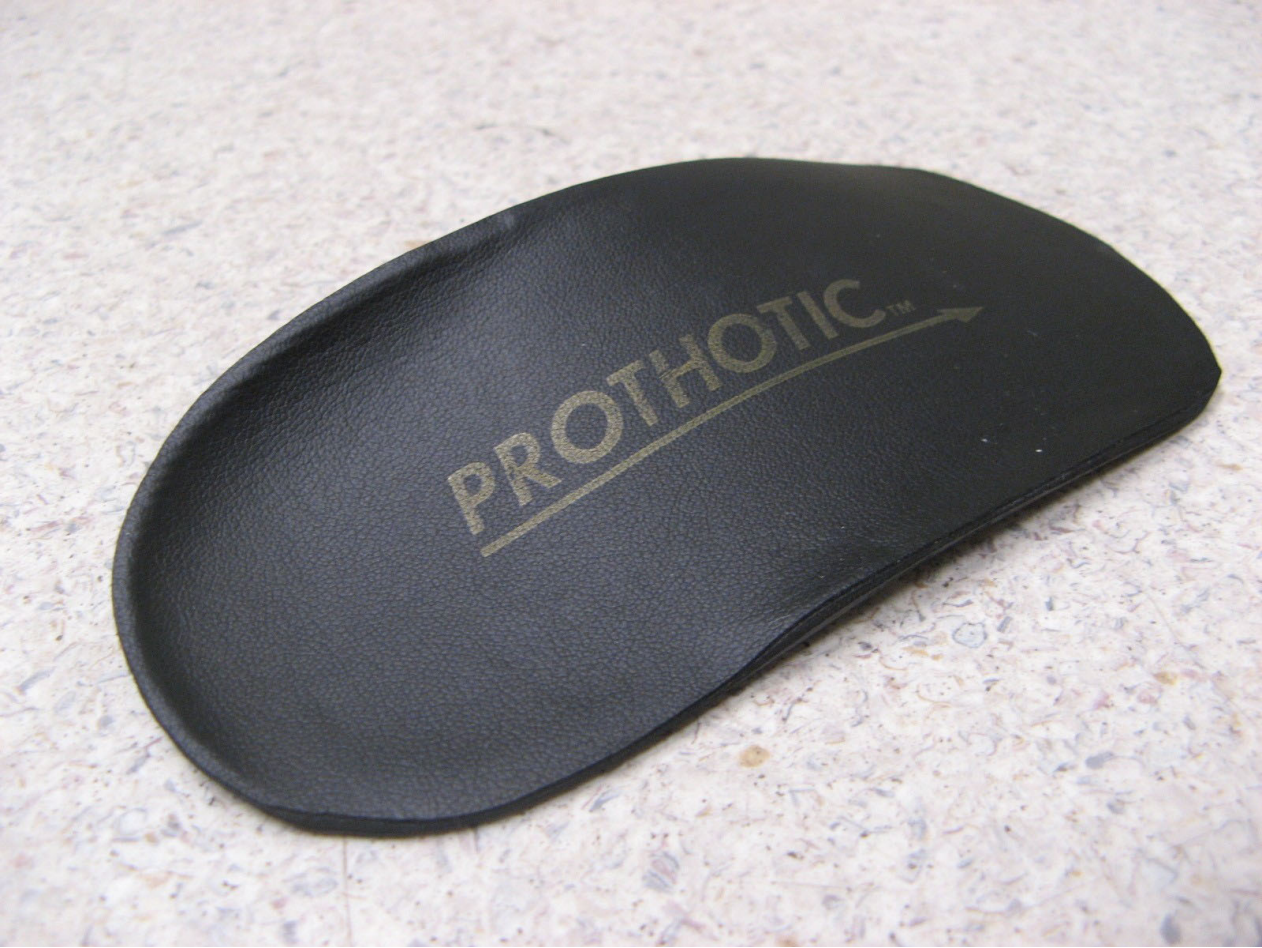
**Heel raise shoe orthoses**.

**Figure 3:**
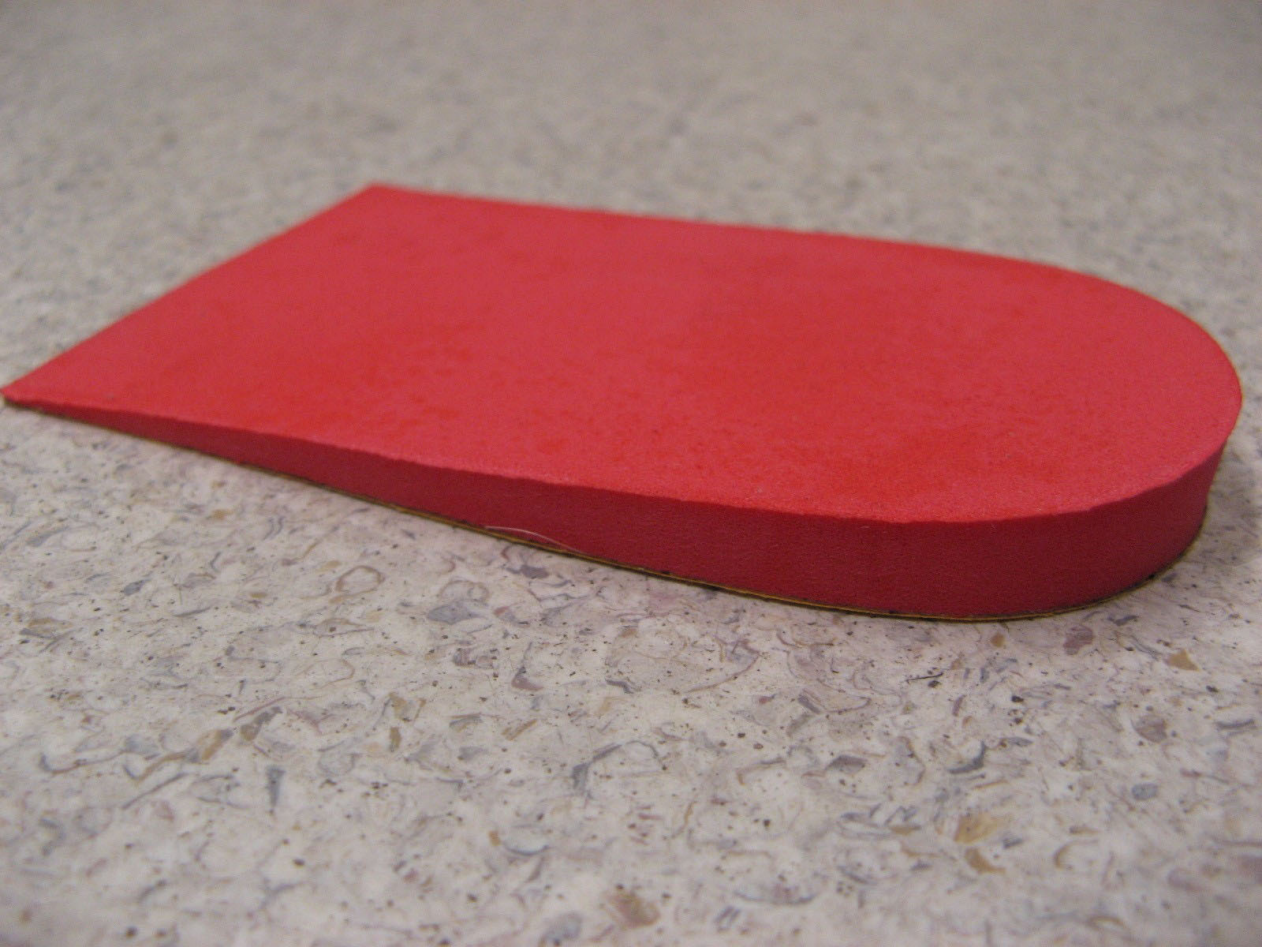
**Prefabricated shoe orthoses**.

The two levels of shoe orthoses to be investigated are:

1. Heel raise (Figure 2)

2. Prefabricated orthoses (Figure 3).

Both of these interventions represent widely available, mutually exclusive treatment approaches that are relatively low in cost compared to customised foot orthoses. Heel raises (Figure 2, 6 mm heel raise) are made from high density ethylene vinyl acetate (EVA). The EVA heel raise is designed to reduce the activity of the gastrocnemius-soleus-achilles tendon complex on the calcaneo-achilles attachment by elevating the calcaneus [33]. Heel raises have been found to provide therapeutic relief in tendoachilles bursitis, tenosynovitis of Achilles tendons, and postoperative management of ruptured Achilles tendons [33]. The prefabricated orthoses (Figure 3, Prothotic: Firm) intervention is a firm prothotic. The prothotic is a polyurethane device that is thought to limit pronation by inverting the rear foot with medial varus wedging combined with a small notch in the cuboid area [34]. The authors have anticipated that the use of a medical varus wedging device is contraindicated with a FPI equal or less than -1. Should the child present with a FPI equal or less then that the child will be excluded from the study and offered alternative treatment through the health service. The orthoses will be covered in a 2 mm blown multi-density EVA cover (Multiform) which is anticipated to provide shock absorption. There is currently no literature on the effectiveness of any custom, semi-custom or prefabricated orthotic device in the treatment of posterior heel pain associated with calcaneal apophysitis [35].

#### Factor 2: Footwear

The two levels of footwear to be investigated are:

1. Current footwear worn by participant

2. New athletic footwear provided by study.

The first condition in this factor entails no direction for modification of current footwear being provided by the treating podiatrist. Participants will be requested to continue wearing their most commonly worn footwear. This may be school shoes, sports shoes or casual shoes dependent on the patient.

The alternate condition is the new athletic footwear prescription/replacement intervention. This involves provision of a shoe with a firm heel counter, dual density EVA midsole and rear foot control provided by adidas Australia. All shoes provided will be the same model. The footwear replacement intervention will be provided to the participant at no cost. All participants within this group will be given standardised shoe wearing in instructions. Schools within the study area allow the students to wear athletic footwear as the chosen footwear style therefore compliance with school uniform, sport and play is not anticipated to be an influencing factor. Should an issue arise; the treating podiatrist will give a letter of support for the footwear choice and liaise with the school if required.

### Instrumentation

The primary outcome measure for this study is the Oxford Foot and Ankle Questionnaire [36]. This scale measures the disability associated with foot and ankle problems in children aged from 5-16 years. This assessment is taken from the perspective of both the child and the parents and contains "physical" (6 items, Cronbach's alpha = 0.92, parent-child intraclass correlation coefficient (ICC) = 0.72), "school and play" (4 items, Cronbach's alpha = 0.89, parent-child ICC = 0.73) and "emotional" (4 items, Cronbach's alpha = 0.86, parent-child ICC = 0.72) domain areas [36].

Secondary outcome measurements will be the Faces pain scale [37,38] and the Lunge Test [38]. The Faces pain scale is a seven point verbal rating scale that will be used to measure severity of pain at rest, on palpation, during activity and after activity (2 hours post) [36,37]. The test-retest reliability data for six-year-old children yielded a rank correlation coefficient of 0.79, indicating that the scores obtained using the faces pain scale are adequately reproducible over time. Inter-rater reliability produced a high rank correlation coefficient of 0.82 [37].

The Lunge Test [39] is a clinical measure of ankle dorsiflexion. All participants will be given a standardised stretching program; measurement of the lunge test will be recorded to determine any change in ankle dorsiflexion. Intra-rater reliability of experienced raters conducting this test has been shown to be high when using a digital inclinometer (average ICC = 0.88, average 95% limits of agreement = -6.6° - 4.8°) and the clear acrylic plate (average ICC = 0.89, average 95% limits of agreement = -7.2° - 4.3°) to assist with measurement [39]. The intra-rater reliability of an inexperienced rater has also been demonstrated to be good to high when using a digital inclinometer (ICC = 0.77, 95% limits of agreement = -9.1° - 8.3°) and a clear acrylic plate apparatus (ICC = 0.89, 95% limits of agreement = -8.1° - 4.6°) to assist in measurement. Inter-rater reliability for inexperienced raters has also been found to be high for when using either the digital inclinometer (ICC = 0.95, 95% limits of agreement = -5.7° - 5.7°) and the clear acrylic plate apparatus (ICC = 0.97, 95% limits of agreement = -4.7° - 4.7°) [39].

Demographic data, including participant age, gender, height standard deviation and weight standard deviation, will be collected for all participants at the baseline assessment along with the Foot Posture Index-6 (FPI-6), a clinical standardised measure of a participant's standing foot posture [40]. This assessment allows for biomechanical factors to be examined, which has been suggested throughout the literature as a possible causative factor of calcaneal apophysitis.

#### Compliance measures

Participants will be asked to complete a star chart or star sticker placement within their school diary to daily log compliance with allocated shoe insert intervention/footwear and record days of ice application and stretching.

### Procedure

All patients presenting to the study locations with heel pain will be screened for study eligibility by their treating podiatrist. The parents of patients who meet the study inclusion criteria will be provided with a written and verbal explanation of the study, and will be asked to provide consent for their child to participate. Those for whom consent to participate is provided will have baseline assessments undertaken prior to randomisation so the assessor is blinded to participant group allocation at this time.

Randomisation will then be undertaken using a permuted-block randomisation approach stratified by site. Randomisation blocks of four or eight participants will be generated and randomly selected and the resultant allocation order will be entered into opaque, sealed envelopes for each site. An investigator not involved in recruitment or assessment of participants (Terry Haines) will be responsible for preparing the random allocation sequence and envelopes. The treatment conditions will be provided as per the random allocation sequence following completion of the initial assessment.

As remote randomisation is not feasible, a set of tamper- evident envelopes will be provided to each participating site. The envelopes will look identical, and each will have the trial indication and a sequential number on it. The envelopes will be opaque and well sealed and the sequence of opening the envelopes will be monitored regularly by a non participating staff member who will be responsible for storing and issuing the concealed allocation envelopes. As there is no off site randomisations there is potential for bias, the authors have attempt to mitigate this concern by having the randomisation kept in a secure location

Primary and secondary outcome measurements will be undertaken at initial presentation and at one, two and three month follow-up appointments, a tolerance of +/- 1 week will be universally applied. These review appointment dates are routinely employed for this patient population and do not represent a departure from standard practice. Pre-appointment reminder text message will be employed to promote re-attendance at follow-up appointments. If a participant does not re-attend a follow-up appointment, the trial podiatrist will telephone the participant to attempt to reschedule the appointment. In the case of non-attendance, the Oxford Foot and Ankle Questionnaire will be posted with a reply-paid, addressed envelope. In the case of non-return of the questionnaire, a telephone consultation will be provided to offer completion of this questionnaire.

If a participant's pain does not resolve in the three month treatment trial an individualised podiatric assessment and treatment will be offered.

### Adverse Events

Adverse events will be measured and recorded during the study. The adverse events may include incidents such as skin reactions (e.g., blisters or rashes) from the prefabricated orthoses or heel lifts.

### Analysis

The intervention factors will be examined over time on the primary and secondary outcome measures using Generalised Estimating Equations. This approach is suitable for analysis of longitudinal data and has been shown to produce unbiased effect estimates with appropriate precision in the presence of missing data (missing completely at random, missing at random or missing not at random) without the need for data imputation techniques and does not involve list wise deletion of participant data where missing data is present [41]. The analysis will be undertaken to examine the main effects of the two intervention factors, however, if a significant "shoe orthoses by footwear" interaction term is identified, simple effects will be focused upon. The analysis will follow the intention-to-treat principle.

A follow-up per-protocol analysis will be conducted to account for participants who do not adhere to their allocated treatment protocol. However, such analyses will be described as being exploratory and will not be the focus of the resulting manuscript

### Sample size

It is considered that a minimum clinically important change in the Oxford Ankle Foot Questionnaire in any domain is 7 points and that based on previous work, the maximum standard deviation in any domain is 6 points [36]. Given this experiment has 1 pre-intervention measure and 3 post-intervention measures, a sample size of n = 27 per factorial trial cell (i.e., total trial n = 108) will have >90% power to detect a significant difference of 7 points in any simple contrasts undertaken, assuming a correlation between assessment points within individual participants is r = 0.7. To account for 15% drop outs and incomplete assessments, a total of n = 124 will be recruited.

### Ethical Consideration

Ethical approval for this study has been obtained by the Southern Health Human Research Ethics Committee HREC Ref: 09271B. Registration of this randomised control trial has been completed with the Australian New Zealand Clinical Trial Registry ACTRN12609000696291.

## Conclusion

Posterior heel pain associated with a clinical diagnosis of calcaneal apophysitis is a common disorder amongst pre-teen children. Despite this, there is presently no randomised controlled trial of clinical treatment options. This trial will provide evidence of the efficacy for some commonly used treatment options from a randomised trial for the first time. The outcomes of this trial are therefore likely to strongly influence practice guidelines and clinical care in this area.

This trial will be limited by its inability to blind outcome assessors (who are the patients' treating podiatrists) though the potential impact of this on trial results is questionable. The primary outcome measure (Oxford Foot and Ankle Questionnaire) is a patient and parent self-report measure, as is the Faces pain scale secondary outcome, hence there may be limited potential for treating podiatrists to influence these results. This trial will be limited by its inability to blind the patients receiving the treatment modalities. This is believed to more likely to affect the new footwear factor, as this compares an active to an inactive treatment level.

This trial is also limited in its ability to test all different combinations of possible treatment factors as there are several other possible treatment approaches available. The present treatment approaches were selected as they were considered by the investigators to be some of the most commonly used strategies available that were also likely to demonstrate clinical efficacy.

A paucity of research evidence supporting the theorised pathomechanics and efficacy of treatment options for a condition such as calcaneal apophysitis creates uncertainty for clinicians attempting to pursue an evidence-based treatment approach. These trials, and other similar trials, are needed to help clinicians better understand this condition and the efficacy of treatment approaches they provide.

## Competing interests

The authors declare that they have no competing interests.

## Authors' contributions

All the authors were involved in the design and conception of the work within this paper. AJ and CW drafted the manuscript with critical revision and ongoing support and advice from TH. All authors read and approved the final manuscript.
